# Targeted Intracellular Delivery of Resveratrol to Glioblastoma Cells Using Apolipoprotein E-Containing Reconstituted HDL as a Nanovehicle

**DOI:** 10.1371/journal.pone.0135130

**Published:** 2015-08-10

**Authors:** Sea H. Kim, Birendra Babu Adhikari, Siobanth Cruz, Michael P. Schramm, Joe A. Vinson, Vasanthy Narayanaswami

**Affiliations:** 1 Department of Chemistry and Biochemistry, California State University Long Beach, Long Beach, California, United States of America; 2 Chemistry Department, Loyola Science Center, University of Scranton, Scranton, Pennsylvania, United States of America; Katholieke Universiteit Leuven, BELGIUM

## Abstract

The objective of this study is to transport and deliver resveratrol to intracellular sites using apolipoprotein E3 (apoE3). Reconstituted high-density lipoprotein (rHDL) bearing resveratrol (rHDL/res) was prepared using phospholipids and the low-density lipoprotein receptor (LDLr)-binding domain of apoE3. Biophysical characterization revealed that resveratrol was partitioned into the phospholipid bilayer of discoidal rHDL/res particles (~19 nm diameter). Co-immunoprecipitation studies indicated that the LDLr-binding ability of apoE3 was retained. Cellular uptake of resveratrol to intracellular sites was evaluated in glioblastoma A-172 cells by direct fluorescence using chemically synthesized NBD-labeled resveratrol (res/NBD) embedded in rHDL/res. Competition and inhibition studies indicate that the uptake is by receptor mediated endocytosis via the LDLr, with co-localization of apoE3 and res/NBD in late endosomes/lysosomes. We propose that rHDL provides an ideal hydrophobic milieu to sequester resveratrol and that rHDL containing apoE3 serves as an effective “nanovehicle” to transport and deliver resveratrol to targeted intracellular sites.

## Background

Resveratrol (3,5,4'-trihydroxystilbene) (Res) is a phytoalexin that belongs to the stilbene family of compounds, which consist of two phenolic rings linked by a styrene double bond. It occurs naturally in grape skins, peanuts and some berries. As a polyphenol it appears to have several beneficial health roles, in addition to its promising biological effects in a wide range of disease states such as cancer, heart disease and diabetes in animal models [1, 2]. It has also been shown to be anti-atherogenic [3–5] and to reduce oxidative stress [6]. Taken together, there has been increased scrutiny of the potential use of resveratrol as a health and well being nutrient in humans.

A general unifying mechanism of action by resveratrol underlying many of the disease states appears to be its role as an anti-inflammatory and/or antioxidant agent [1, 7], and through activation of Sirt1[8, 9], the NAD^+^-dependent protein deacetylase, which regulates a wide variety of signaling pathways. Resveratrol exists in the *cis*- and *trans*- forms, with the *trans*-isomer being more biologically active (unless otherwise specified, the term resveratrol refers to *trans*-resveratrol throughout the text). It is poorly soluble in aqueous system; in humans, orally ingested resveratrol is absorbed either by diffusion or through ATP-dependent binding cassette transporters [10] and rapidly eliminated [11]. The plasma concentration of resveratrol is typically low, with the hepatic conversion to the relatively polar glucuronidated and sulfated derivatives[12–15] being a major contributory factor. Its metabolites therefore remain mainly in the gastrointestinal tract, and may not be as effective as anti-oxidants as their unmodified form. Several different approaches have been adopted to increase the maximal plasma resveratrol concentration and its bioavailability by increasing dosage [16], co-administering biomolecules that target and inhibit critical enzymes involved in conjugation and modification [17], or administering its precursor form that can be potentially converted by the body to the active form [18, 19]. Recent studies have directed their attention to nanoscience as an alternative approach to transport and deliver resveratrol in its potent unmodified state (in the absence and/or presence of additives) at the required sites at the tissue and cellular level. Polymer- and lipid-based nanoparticles and nanocapsules, or cyclodextrin-based nanosponges have been formulated to incorporate resveratrol and increase its aqueous solubility and bioavailability [20–22]. In the current study we investigated the use of the non-polar environment of reconstituted high-density lipoproteins (rHDL) containing apolipoprotein E3 (apoE3) and phospholipids as a possible approach to solubilize resveratrol.

ApoE3 is an anti-atherogenic protein that plays a significant role in plasma cholesterol homeostasis [23, 24]. It is considered anti-atherogenic mainly because of its ability to act as a ligand and mediate cellular uptake of lipoproteins via the low density lipoprotein receptor (LDLr) family of proteins, thereby lowering plasma lipid levels. Lipid-free apoE3 is organized into a 24 kDa N-terminal (NT) domain (residues 1–191) and a 10 kDa C-terminal domain (residues 201–299) [25]. Isolated apoE3-NT domain shows LDLr binding ability that is comparable to that of the intact protein [25]. The LDLr binding ability of apoE3 is elicited mainly in the lipid bound state [25].

Our current understanding of the structure of the lipid-associated state of apoE3 is based on spectroscopic and biophysical data of rHDL, which are composed of a bilayer of phospholipids held together by a “double belt” of apoE3 in an extended helical organization [26]. These are large (~ 600 kDa), discoidal (15–20 nm diameter) water-soluble lipoprotein complexes that resemble nascent HDL generated *in vivo*. The lipid bilayer offers an excellent environment to harbor hydrophobic compounds that can be embedded, and therefore shielded, from the aqueous environment. In this study, we report the use of rHDL to transport and deliver resveratrol to intra-cellular sites by receptor-mediated endocytosis using the NT domain of apoE3 as a ligand to bind cell surface localized LDLr in glioblastoma cells.

## Methods

*Trans*-resveratrol (98+% pure), 4-Chloro-7-Nitrobenz-2-Oxa-1,3-Diazole (NBD) and 16 DOXYL-stearic acid (16-DSA) were purchased from Sigma Aldrich (St. Louis, MO), potassium iodide (KI) and sodium thiosulfate from Fisher Scientific (Fair Lawn, NJ), and 1,2-dimyristoyl-*sn*-glycero-3-phosphocholine (DMPC) from Avanti Polar Lipids (Alabaster, AL). Phospholipid assay kit was from Wako Chemicals USA, Inc. (Richmond, VA), DC and BCA kit for protein assay from BioRad Laboratories (Hercules, CA). Human brain A-172 glioblastoma cells were obtained from ATCC (Manassas, VA), while DMEM, fetal bovine serum (FBS) and lipoprotein deficient serum (LPDS) were from Life Technologies (Grand Island, NY). All solvents used were of analytical grade.

### Expression, isolation and purification of apoE3-NT

Recombinant human apoE3-NT domain bearing residues 1–191 (apoE3-NT) and a hexa His-tag was purified as described earlier [27]. Protein concentration was determined based on the molar extinction coefficient for apoE3(1–191) at 280 nm (27,960 M^-1^ cm^-1^).

### Reconstitution of HDL with resveratrol

rHDL containing DMPC and apoE3-NT (5:2 w/w ratio) was prepared by the sonication method using 20 mM sodium phosphate, pH 7.4 containing 150 mM NaCl (phosphate buffered saline, PBS) as described previously,[28] in the absence or the presence of resveratrol. The starting ratio of lipid: protein: resveratrol was 5:2:5 (w/w). Since resveratrol was dissolved in DMSO, control samples of rHDL without resveratrol had DMSO alone (<5% v/v). The samples were incubated at 24°C for 16 h, followed by KBr density gradient ultracentrifugation (230,000 x g for 5.5 h). Fractions containing co-localized protein and phospholipid were pooled (Fig A in S1 File), dialyzed against PBS. The two preparations (without and with resveratrol) are designated as rHDL and rHDL/res, respectively. The details of the density gradient ultracentrifugation results are provided in S1 File.

### Spectroscopic characterization of rHDL and rHDL/res

The presence and location of resveratrol in rHDL/res were determined based on its intrinsic fluorescence properties. Initially, UV-Vis spectra of rHDL and rHDL/res were recorded from 200 to 500 nm (UV-2401 SHIMADZU spectrophotometer) in PBS and compared with that of 0.2 μM resveratrol in DMSO, isopropanol, ethyl acetate, 95% ethanol, or water. Steady state fluorescence spectra of rHDL and rHDL/res were recorded between 320 and 450 nm at 24°C following excitation at 310 nm, at 50 nm/min with 3.0 nm excitation and emission slit widths (Perkin-Elmer LS55B fluorometer).

Fluorescence quenching by KI was carried out by addition of small increments of stock solutions (0.04, 0.4, 4, and 6 M) (containing 1 mM sodium thiosulfate to prevent formation of free iodine) to rHDL/res (70 μg/ml protein). Quenching with 16-DSA was performed as above by addition of stock solutions (0.125, 2.5, 25 and 250 mM) in DMSO, maintaining the final volume of DMSO at ≤ 5% v/v). Fluorescence emission intensities were recorded at 384 nm following excitation at 310 nm. Quenching data were analyzed using the Stern-Volmer equation, F_0_ /F = 1 + K_SV_ [Q], where F_0_ and F represent the fluorescence intensities in the absence and presence of quencher, respectively, Q is the quencher concentration and, K_SV_ is the apparent quenching constant [29, 30].

### Determination of rHDL/res particle size, diameter, geometry and composition

To determine the size of rHDL/res, non-denaturing PAGE was carried out using 4–20% acrylamide gradient (loading~ 50 μg protein sample). Electrophoresis was carried out in the presence of protein standard markers (Amersham HMW Calibration Kit, G.E. Healthcare) for 18 h at 132 V at 4°C, and the gels stained with 0.5% Amido Black. The particles were visualized by transmission electron microscopy (TEM) operating at 90 keV (JEOL 1200 EX ΙΙ electron microscope) following negative staining with 2% sodium phosphotungstate. Particle composition was determined based on protein, phospholipid and resveratrol concentration (the latter by RP-HPLC, using resveratrol in sterile water as standard (Fig B in S1 File). In all cases, rHDL was used as a control.

### Co-immunoprecipitation (co-IP) assay to assess LDLr binding activity of rHDL/res

To examine the LDLr binding ability of rHDL/res, a co-IP assay was carried out as described previously [31, 32] using a construct bearing the soluble LDLr ligand binding domains LA3–LA6 with *c-Myc* epitope (sLDLr). Briefly, 10 μg of sLDLr was incubated with rHDL/res or rHDL (10 μg protein) in the presence of 2 mM Ca^2+^ in PBS at 4°C for 1 h, followed by co-IP with an anti-*c-Myc* antibody-linked agarose to capture the rHDL/sLDLr or rHDL/res/sLDLr complexes. ApoE3 was detected by Western blot analysis using HRP-conjugated polyclonal apoE antibody. A replica experiment was conducted wherein an anti-*c-Myc* antibody (9E10) was utilized to identify the presence of LDLr in each reaction.

### LDLr-mediated binding and uptake of rHDL/res by glioblastoma cells

LDLr mediated uptake of rHDL/res was determined using human glioblastoma cell line A-172. The cells were cultured in DMEM with 10% FBS in presence of 5000 IU/mL penicillin and 5000 μg/mL streptomycin sulfate at 37°C according to ATCC guidelines. For uptake experiments, the cells were grown ~60% confluency on a cover glass, placed in a 6-well cell culture cluster (with ~ 1x10^6^ cells per well). They were then washed with pre-warmed medium containing 10% LPDS and incubated for 24 h to induce LDLr expression. Cellular uptake of apoE3 was followed by immunofluorescence using a mAb1D7, and Alexa555-labeled secondary antibody. Uptake of the lipid components was visualized by direct fluorescence using rHDL/res or rHDL containing 1% 1,1'-dioctadecyl-3,3,3',3'-tetramethylindocarbocyanine iodide (DiI, Invitrogen Life Technologies, Grand Island, NY). About 50 μl of a stock solution of DiI (0.33 mg/ml) in DMSO was incubated with rHDL/res or rHDL at 37°C for 18 h in dark. Unbound DiI was separated from lipoprotein-bound DiI by density gradient ultracentrifugation. The top fractions containing DiI labeled rHDL/res (rHDL/res/DiI) or rHDL (rHDL/DiI) were pooled together and dialyzed. Uptake of resveratrol was visualized by direct fluorescence using NBD-labeled resveratrol (res/NBD). About 5% res/NBD was included with unlabeled resveratrol for incorporation into rHDL during the reconstitution procedure to generate rHDL/res/NBD.

All unlabeled and labeled rHDL/res and rHDL samples were passed through a 0.22 μM filter prior to addition to cells. The cells were incubated with 5 μg of unlabeled rHDL/res or rHDL or rHDL/res/NBD in 10% LPDS for 1–3 h at 37°C to follow uptake. In control experiments, the cells were treated with an equivalent amount of res/NBD in DMSO (as assessed by fluorescence emission intensity of res/NBD) to evaluate the uptake of resveratrol in the absence of carrier vehicle. The cells were washed 3 times with pre-warmed Dulbecco’s PBS (DPBS), fixed with 3.7% formaldehyde and permeabilized with 0.2% Triton X-100 for 5 min at 37°C. They were stained with DAPI (4',6-Diamidino-2-phenylindole dihydrochloride) in DPBS to visualize the nucleus. Cellular localization of internalized rHDL/res/NBD after 2 h of incubation with cells was determined using anti-late endosomal/lysosomal marker, Lysosome Associated Membrane Protein 1 (LAMP1) (D2D11) XP rabbit monoclonal antibody (1:100) and Alexa Fluor 594 conjugated anti-rabbit IgG (1:200) (Cell Signaling Technology, Inc., Danvers, MA).

In independent experiments the cells were incubated with unlabeled or labeled rHDL/res in the presence of 50-fold excess human LDL (w/w) (Sigma Aldrich, St. Louis, MO) or 2 mM suramin for 3 h at 37°C. The cells were visualized by confocal laser scanning microscopy (Olympus IX-81) and the images captured via Olympus Fluoview 1000.

### Synthesis of res/NBD

Res/NBD was synthesized using commercially available materials. The details of the synthesis are provided in S1 File.

## Results

The details of rHDL preparation in the absence and presence of resveratrol (rHDL and rHDL/res, respectively) and separation of rHDL from lipid-free apoE3-NT are described under S1 File, and Fig A in S1 File.

**Fig 1:**
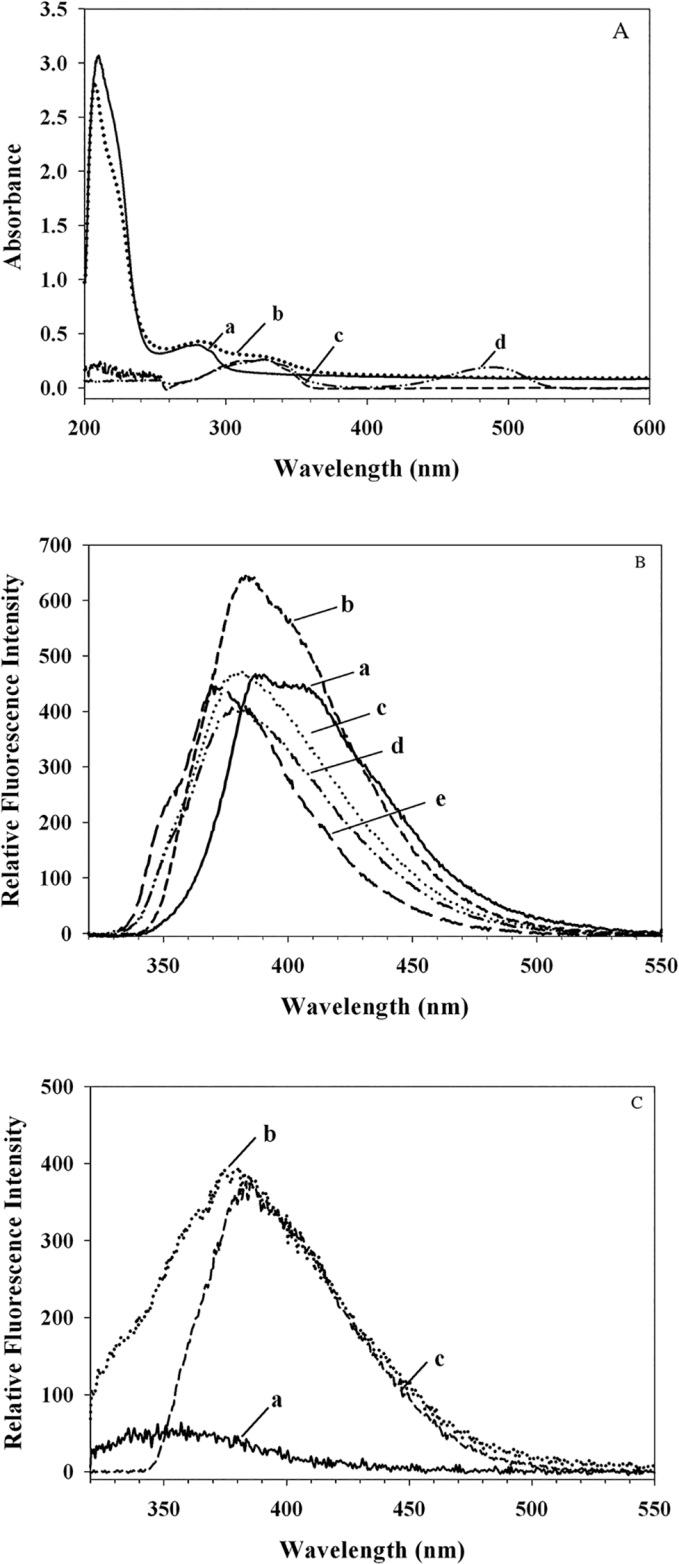
Spectra characteristics of rHDL and rHDL/res. (**A**) The absorbance spectra of pooled fractions of rHDL (a), rHDL/res (b), resveratrol in DMSO (c) and res/NBD in DMSO (d) were recorded. (**B**) Fluorescence emission spectra of resveratrol were recorded in: water (**a**), DMSO (**b**), 95% ethanol (**c**), isopropanol (**d**), and ethyl acetate (**e**) with dielectric constants of 80.4, 33.0, 24.3, 7.5 and 6.0, respectively, following excitation at 310 nm. (**C**). Fluorescence emission spectra of rHDL (**a**) and rHDL/res (**b**) were recorded with 10 μg protein in PBS. For comparison, emission spectrum of resveratrol in DMSO is shown (**c**).

The absorbance spectra (Fig 1A) of rHDL and rHDL/res were compared with that of resveratrol in DMSO. Compared to the spectrum of rHDL, which reveals only protein absorbance at 280 nm (*Spectrum a*), that of rHDL/res reveals an additional peak at ~ 330 nm (S*pectrum b*), similar to absorbance of resveratrol in DMSO (*Spectrum c*).

We exploited the intrinsic fluorescence properties of resveratrol to determine its presence in rHDL. In initial experiments the fluorescence emission of resveratrol was characterized in solvents of varying polarity, as reflected by their dielectric constants. Fig 1B shows that the λ_max_ elicits a blue shift from 390 to ~370 nm as the solvent polarity decreases. This environment-dependent shift in the λ_max_ of resveratrol was used to determine its location with respect to the lipoprotein complex in subsequent experiments. The fluorescence emission spectrum of rHDL/res elicited significant fluorescence intensity with λ_max_ at ~ 382 nm (Fig 1C, *spectrum b*), comparable to that of resveratrol in DMSO (Fig 1C, *spectrum c*). This suggests that the microenvironment of resveratrol in rHDL is relatively hydrophobic with a polarity similar to that of DMSO. In contrast, rHDL showed low fluorescence emission at wavelengths >370 nm (Fig 1C, *spectrum a*); the small peak at ~ 350 nm is possibly due to resonance energy transfer between Trp/Tyr residues in apoE3-NT and resveratrol in rHDL/res. Together, the spectroscopic measurements indicate the presence of resveratrol in the pooled lipoprotein-containing fractions.

**Fig 2:**
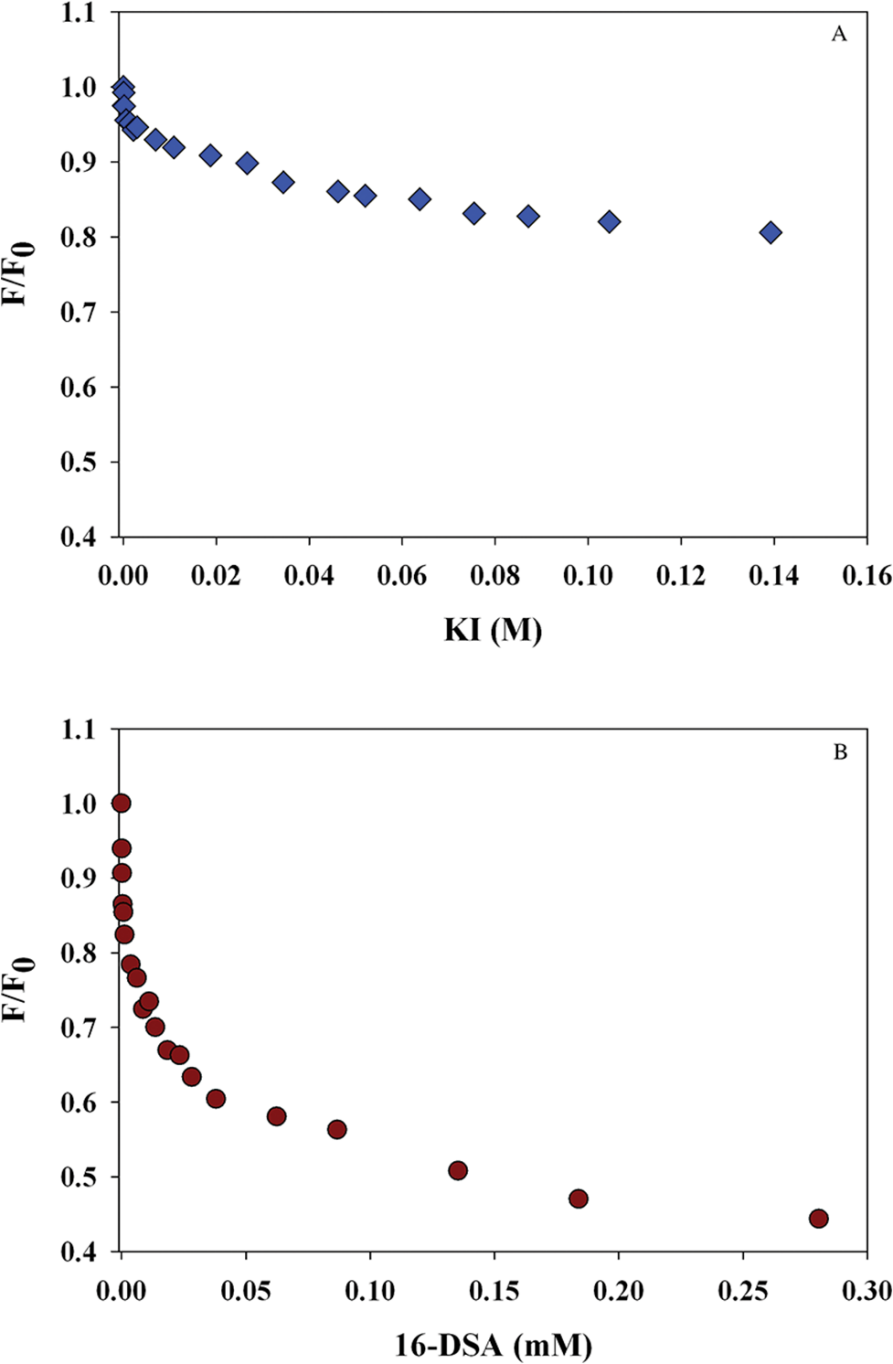
Quenching analysis of rHDL/res. rHDL/res (10 μg protein) was treated with increasing concentrations of KI in PBS (**A**) or 16-DSA in DMSO (**B**), and the fluorescence emission intensity recorded at each concentration. Data are plotted as F/F_0_ versus quencher concentration. Representative data from 3 independent experiments are shown.

To further investigate the location of resveratrol in rHDL, its fluorescence emission was subjected to quenching by KI and 16-DSA, aqueous and lipid based quenchers, respectively. The rationale is that the quenching pattern would aid in distinguishing between resveratrol’s location in the hydrophobic core of the lipoprotein complex and the polar exterior facing the aqueous environment. Fig 2A shows the plot of F/F_0_ versus [KI], resulting in a quenching constant of 4.29 M^-1^ (obtained from the initial slope of the Stern-Volmer plot at low quencher concentrations). On the other hand, a significant decrease in F/F_0_ was noted with increasing concentration of 16-DSA (Fig 2B), yielding an apparent quenching constant of 6.01 x 10^−2^ M^-1^. It should be noted that when using spin-labeled fatty acids as quenchers, the classic Stern-Volmer equation for free diffusion does not apply since the quencher is not uniformly distributed in solution over the corresponding space coordinate [33, 34] but restricted within the space of the lipoprotein particles; therefore it necessitates calculation of an apparent quenching constant. Thus although a direct comparison of the quenching constants for KI and 16-DSA is not possible, it is relevant to note that 16-DSA was a far more powerful quencher of resveratrol fluorescence compared to KI. This indicates that resveratrol is located deep in the hydrophobic milieu of the phospholipid bilayer of the lipoprotein complex, which is consistent with the highly blue-shifted λ_max_ of resveratrol in rHDL/res samples.

**Fig 3:**
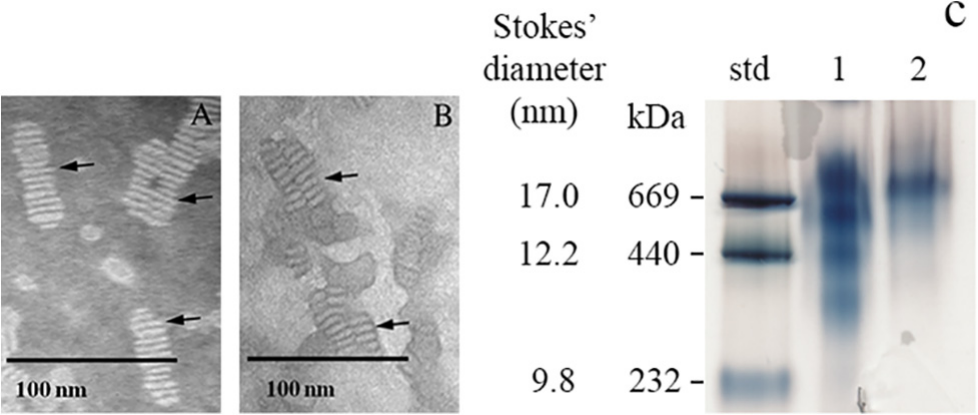
Characterization of rHDL and rHDL/res particles. **A & B. Transmission electron microscopy**. Negative staining of rHDL (**A**) and rHDL/res (**B**) was carried with 10 μg protein. The bar represents 20 nm. Arrows draw attention to discoidal particles. **C. Non-denaturing PAGE**. rHDL and rHDL/res were electrophoresed on 4–20% acrylamide gradient gel. The far left lane bears the high molecular mass standards; the molecular mass and their corresponding Stokes’ diameters are indicated; lane 1) rHDL, and lane 2) rHDL/res. Arrows draw attention to particle heterogeneity in lane 1.

In the next step, TEM was used to visualize the geometry and size of rHDL/res in comparison with rHDL (Fig 3A and 3B). The rHDL/res particles (**B**) were discoidal in shape similar to that noted for rHDL (**A**) suggesting that the presence of resveratrol did not alter the overall shape of the lipoprotein complexes. The average diameter of rHDL was 17.4 ± 0.2 nm (n = 75) with sizes ranging from ~ 12 nm to ~ 20 nm. The average diameter of rHDL/res particles was 19.2 ± 0.02 nm (n = 69). Further, non-denaturing PAGE analysis (Fig 3C) indicates that rHDL/res particles bear an apparent molecular mass of ~ 750 kDa corresponding to ~19 nm diameter, whereas rHDL samples revealed significant heterogeneity (see arrows).

The phospholipid and protein composition of rHDL/res were 2.68 mg/ml (3.94 mM) and 3.84 mg/ml (138 μM), respectively, yielding a lipid: protein molar ratio of 29:1. The corresponding concentrations for rHDL were 3.47 mg/mL (5.1 mM) and 3.75 mg/mL (134 μM), respectively (lipid: protein molar ratio of 38:1). The amount of resveratrol in the rHDL/res was determined to be 167 μM by RP-HPLC (Fig B in S1 File). The final lipid: protein: resveratrol ratio in rHDL/res was calculated to be ~30:1:1.

**Fig 4:**
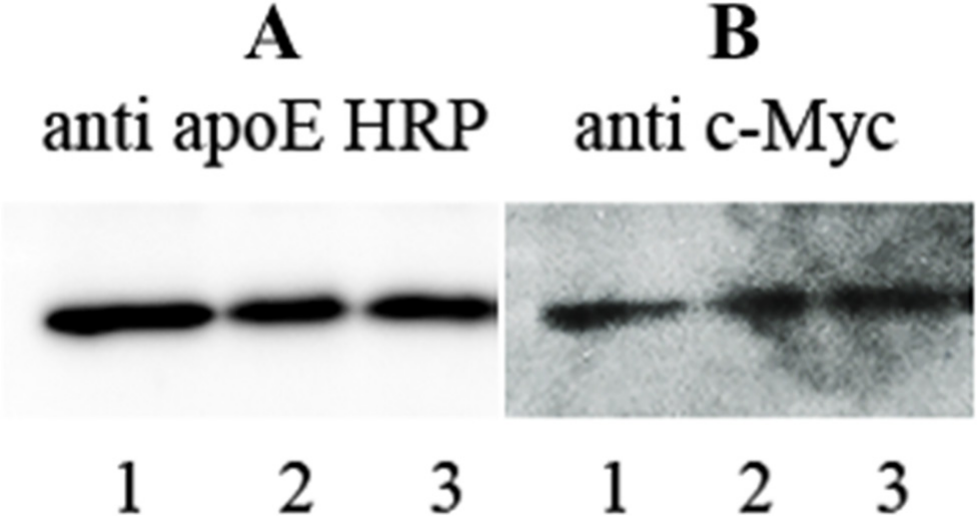
Effect of resveratrol on LDLr binding activity of apoE3. rHDL, rHDL/res or rHDL/res/NBD (10 g protein) was incubated with 10 μg of sLDLr, followed by co-IP with anti-c-Myc-Agarose. sLDLr-bound apoE was detected by Western blot using HRP-conjugated polyclonal apoE antibody (**A**). The corresponding blot using anti-c-Myc antibody is shown for comparison (**B**). The lane assignments are: Lane 1, rHDL; lane 2, rHDL/res; lane 3, rHDL/res/NBD.

To determine if the presence of resveratrol in rHDL affects the LDLr binding ability of apoE3-NT, co-IP was carried out using sLDLr bound to anti-*c-Myc* agarose [31]. Following incubation of rHDL or rHDL/res with sLDLr, the receptor-bound complexes were captured by anti-*c-Myc* bound to agarose and detected by HRP conjugated polyclonal apoE antibody, Fig 4A, or anti-*c-Myc* antibody, Fig 4B. The data show that the presence of resveratrol does not alter the LDLr binding ability of apoE3 in rHDL/res (lane 2).

**Fig 5:**
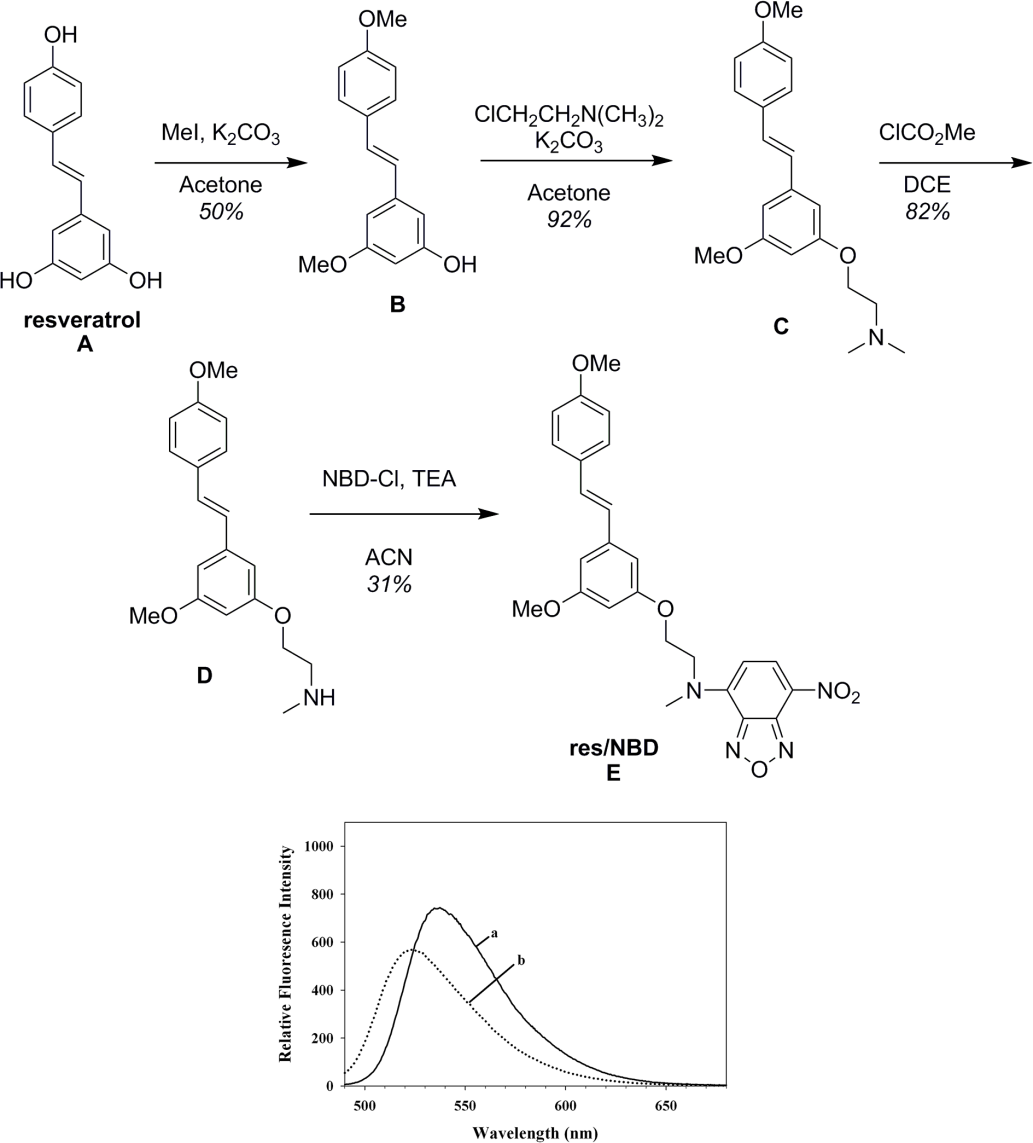
Synthesis scheme and fluorescence emission spectra of res/NBD. To visualize cellular uptake of res, res/NBD was synthesized (*Top*) as described in S1 File. Purified res/NBD was incorporated into rHDL as described under *Methods*. Comparison of the fluorescence emission spectra (*Bottom*) of res/NBD (a) with rHDL/res/NBD (b) shows a blue shift in λ_max_ of NBD, indicative of incorporation of res/NBD in the lipid milieu of rHDL.

To enable direct visualization of cellular uptake of resveratrol, NBD-labeled derivative of resveratrol (res/NBD) was synthesized (Fig 5, *Top* **)**. NBD is significantly lipophilic compared to other green fluorophores such as fluorescein [35], with its lipophilicity comparable to that of resveratrol. Briefly, the synthesis involved statistical protection of 2 of the 3 free phenolic groups by alkylation of resveratrol, **A**, with methyl iodide to give intermediate **B,** Fig 5. This allowed us to insert an ethylene amine functional group on the free phenolic group for subsequent reaction with NBD. Intermediate **B** was reacted with 2-chloro-N,N-dimethylethyleneamine to give **C**, followed by N-demethylation to give **D.** Finally, reaction with NBD-Cl gave **E**, 5-ethoxy-(2-N-methyl-4-amino-7-nitrobenzofurazan)-3,4'-dimethoxy-(Z)-stilbene (res/NBD) in acceptable yield (31%). (REF1: U.S. Provisional Patent Application Serial No. 62/077,780 Filed: November 10, 2014; Our Reference No.: 1958937.00002. REF2: Birendra Babu Adhikari, Sahar Roshandel, Ayu Fujii).

The final product, res/NBD, was characterized by NMR (Figs C, D and E in S1 File) and mass spectrometry (Fig F in S1 File); it was found to retain the main structural features of resveratrol and was obtained in sufficient yields for subsequent uptake studies. The absorbance spectrum of res/NBD in DMSO (Fig 1A, *spectrum d*) reveals peaks at ~330 nm and 480 nm, corresponding to resveratrol and NBD, respectively. Excitation of res/NBD in DMSO at 480 nm results in fluorescence emission with λ_max_ at ~ 540 nm, which is characteristic of NBD (Fig 5, *Bottom*, *spectrum a*). Following reconstitution of res/NBD into rHDL/res, the emission spectrum of rHDL/res/NBD (*spectrum b*) revealed a blue shift in λ_max_ by ~15 nm indicative of a hydrophobic environment. Further, the presence of res/NBD did not alter the LDLr binding ability of rHDL/res/NBD (Fig 4A, lane 3).

The cellular uptake of rHDL/res/NBD was assessed in glioblastoma cells and compared with that of rHDL. Specifically, the experiments were designed to: (i) test the effect of the presence of resveratrol in rHDL on the cellular uptake of lipid and apoE3-NT; (ii) directly compare uptake of res/NBD versus rHDL/res/NBD; (iii) determine sub-cellular localization of resveratrol delivered via rHDL; and, (iv) investigate the uptake mechanism of rHDL/res.

**Fig 6:**
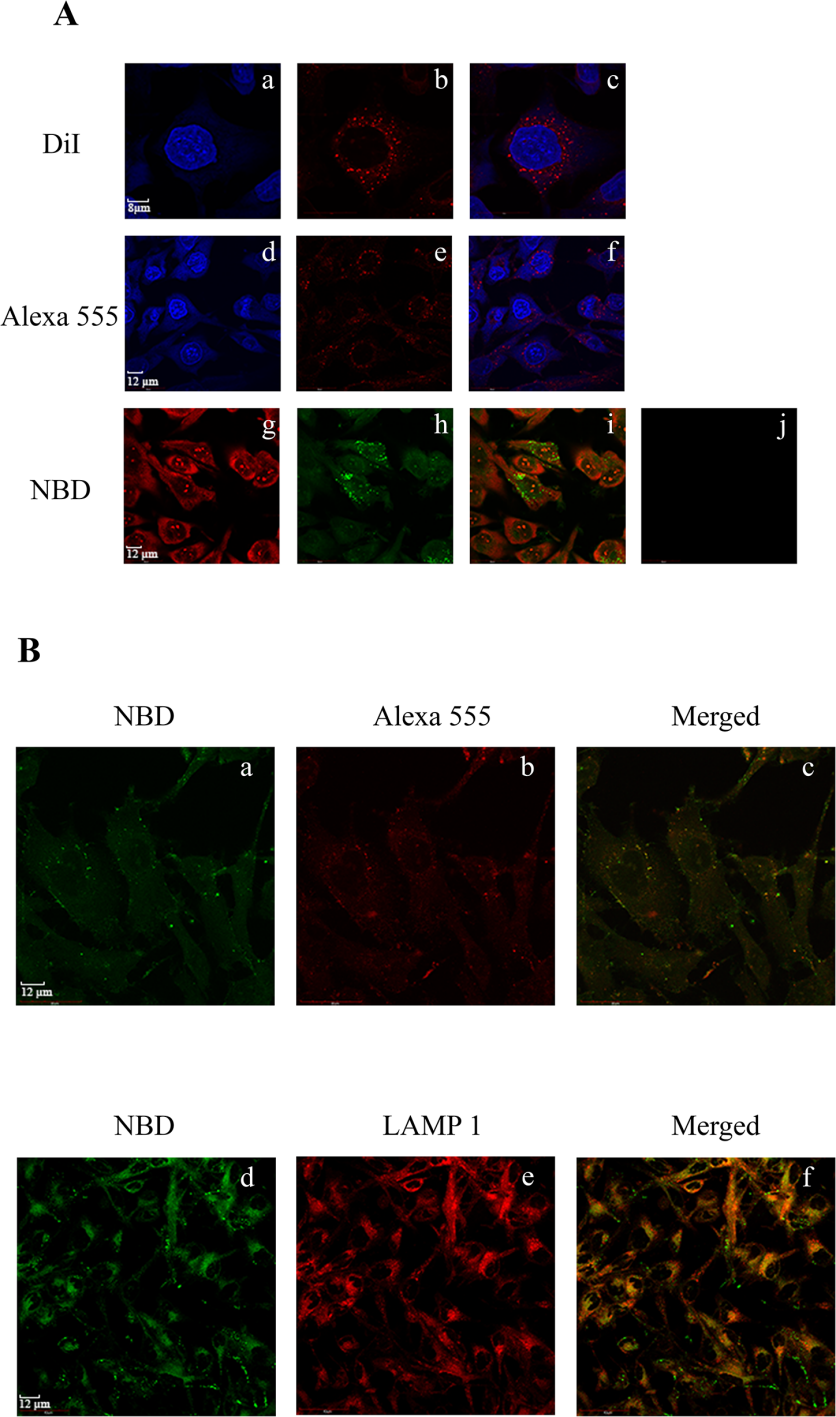
Uptake of rHDL/res by glioblastoma cells. **A**. **Representative confocal images of glioblastoma cells showing uptake of rHDL/res.** Uptake of individual components of rHDL/res was monitored by direct or indirect immunofluorescence: lipid (*a-c*), apoE3 (*d-f)* and resveratrol (*g-i*). Following exposure to rHDL/res/DiI at 37°C for 3h (*a-c*), the cells were visualized under a confocal laser scanning microscope: *a*, DAPI; *b*, DiI; *c*, merge of *a* and *b*. Following exposure to rHDL/res under same conditions (*d*—*f*), the cells were visualized by: *d*, DAPI; *e*, apoE3 monoclonal antibody, 1D7, and Alexa555-conjugated secondary antibody; *f*, merge of *d* and *e*. Following exposure to rHDL/res/NBD (5 μg) as described above (*g*—*i*), the cells were visualized by: *g*, Propidium iodide; *h*, NBD; *i*, merge of *g* and *h*. Panel *j* shows that uptake of res/NBD in the absence of rHDL. **B. Co-localization of res/NBD with apoE3, or with LAMP1 in late endosomal/lysosomal vesicles following cellular uptake of rHDL/res/NBD**. Following exposure to rHDL/res/NBD, the cells were visualized by fluorescence associated with: *a*, NBD to detect res, *b*, Alexa555-conjugated secondary antibody to detect apoE3; *c*, merge of *a* and *b*; *d*, NBD to detect res; *e*, Alexa 594-conjugated secondary antibody to detect LAMP1; *f*, merge of *d* and *e*.

The effect of the presence of resveratrol in rHDL on the cellular uptake of lipids was determined by incubation of cells with DiI labeled rHDL/res (rHDL/res/DiI), Fig 6A, *a*—*c*), which allows direct visualization by confocal microscopy. Internalization of rHDL/res/DiI was inferred from the punctate peri-nuclear endocytic vesicles, similar to that seen if incubated with DiI-labeled rHDL or LDL. The presence of resveratrol in rHDL did not affect the uptake of apoE3 as well, as inferred by immunofluorescence (Fig 6A, *d*—*f*) since a similar punctate pattern was noted in the absence of resveratrol.

The cellular uptake of res/NBD incorporated in rHDL was then compared with uptake of res/NBD alone in the absence of carrier vehicle (Fig 6A, *g*—*j*). While no res/NBD fluorescence was detected in the absence of carrier vehicle under the conditions employed, a significant amount of fluorescence was seen as punctate vesicles when res/NBD was delivered as rHDL/res/NBD. Further, the internalized res/NBD was co-localized with apoE3 (Fig 6B, *a*—*c*) and with LAMP1 (Fig 6B, *d*—*f*) the late endosomal/lysosomal marker. These results suggest receptor-mediated uptake of res/NBD delivered via apoE3-coated rHDL.

**Fig 7:**
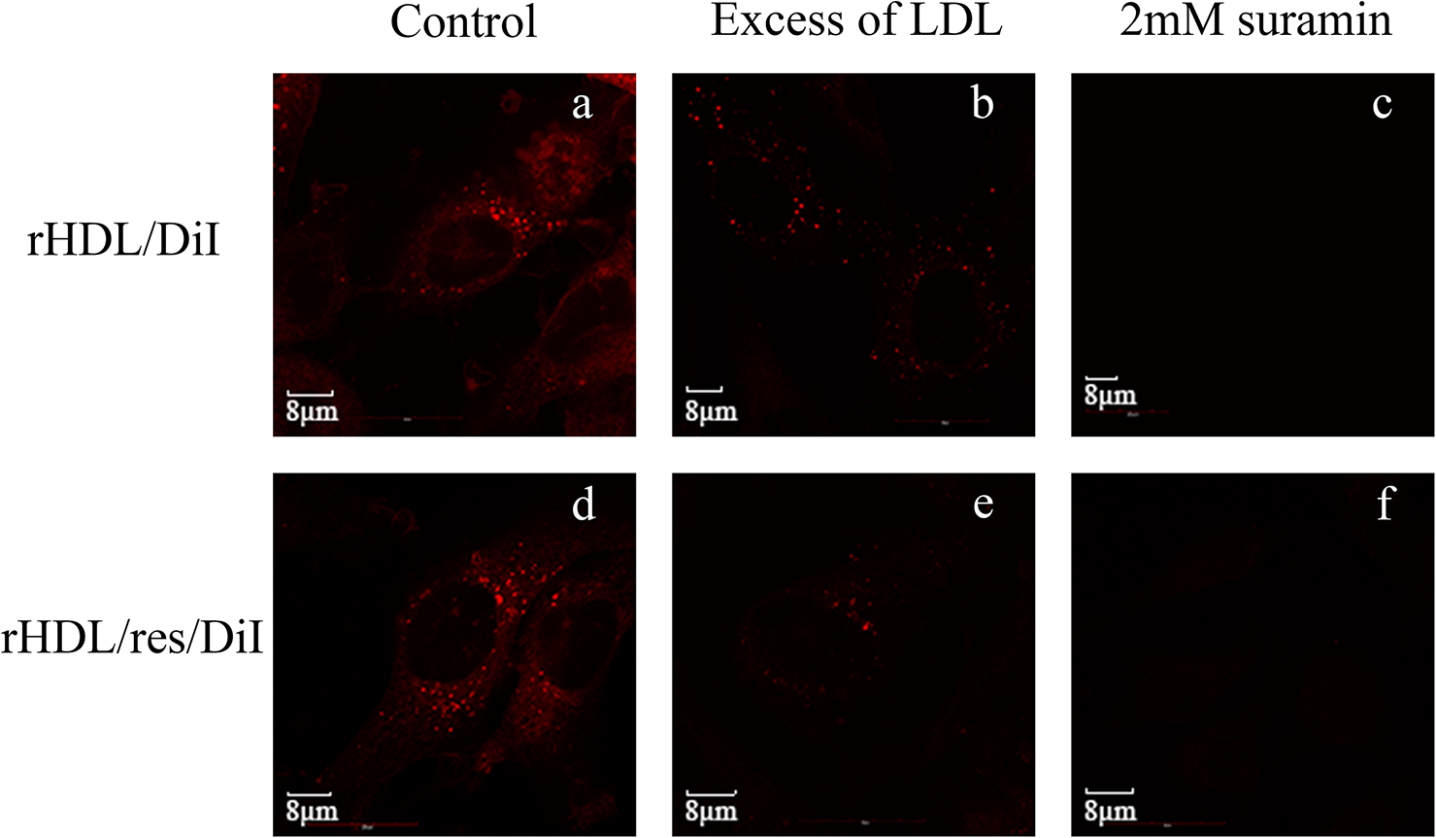
Confocal microscopy images of LDLr-mediated cellular uptake of rHDL/res. Glioblastoma cells were treated with rHDL/DiI (*Top*) or rHDL/res/DiI (*Bottom*) in the absence (*a* and *d*) or the presence of 50-fold excess LDL (w/w) (*b* and *e*) or 2 mM suramin (*c* and *f*) at 37°C. for 3 h. The cells were visualized by the DiI fluorescence associated with lipids.

To confirm that the LDLr mediates the uptake, rHDL/res/DiI was incubated in the absence or presence of excess LDL (1:50 ratio (w/w)), a competitive inhibitor of apoE3 binding to the LDLr, (Fig 7, *d* and *e*, respectively). This was compared to the uptake of rHDL/DiI in the absence and presence of excess LDL (Fig 7, *a* and *b*, respectively). A significant reduction in DiI fluorescence was noted in the presence of LDL, indicative of the involvement of the LDLr. Lastly, the uptake of both rHDL and rHDL/res/DiI were significantly reduced in the presence of suramin, an inhibitor of LDLr binding [36] (Fig 7, *e* and *f*, respectively).

## Discussion

Our study demonstrates that rHDL serves as an effective vehicle to transport resveratrol across the cellular membrane to endocytic sites via LDLr mediated pathway. ApoE3 was the apolipoprotein of choice to reconstitute the rHDL since it serves as a ligand for the LDLr. Other studies have used rHDL-bearing apoAІ (nanodiscs), [37] which do not have the ability to bind and undergo receptor mediate endocytosis with the LDLr.

Fluorescence analysis confirmed that resveratrol was located in a very hydrophobic environment based on the highly blue-shifted λ_max_. Interestingly, the emission spectrum of rHDL/res resembles that of resveratrol in DMSO, with a shoulder at ~400 nm in addition to the major 380 nm peak. The shoulder is also noted for resveratrol in water (Fig 1B, a). This could represent a sub-population of resveratrol that may be located at the protein-lipid interface in rHDL/res or in a second population of lipoprotein particles. The likelihood that it is due to *cis*-resveratrol (that could have arisen from resveratrol by photo-isomerization and/or due to pH alterations [38]) is low since other groups show that the *cis* form absorbs at ~260 nm and emits at 364 and 382 nm [39]. The hydrophobic location of resveratrol in rHDL/res was also inferred from the 70-fold higher apparent quenching constant for fluorescence quenching by 16-DSA compared to that by KI. 16-DSA was an excellent quencher of resveratrol fluorescence since the fatty acid inserts into the lipid bilayer with the hydrophobic tail bearing the quenching moiety facing inward. If resveratrol were to be located on the external surface of rHDL facing the aqueous environment, its fluorescence would be quenched with relative ease by KI. Together, our data indicate that resveratrol is shielded from the aqueous environment by the phospholipid microenvironment and the surrounding protein. This sequestration in the hydrophobic environment may allow the system to achieve a higher resveratrol concentration and increase its bioavailability. Our results are consistent with other reports that show efficient binding of resveratrol to plasma proteins such as albumin and lipoproteins [40, 41], and provide experimental evidence for cellular entry by receptor-mediated endocytosis via the LDLr, as suggested previously [41, 42].

Previous studies have indicated that rHDL contain 4–6 apoE3-NT circumscribing the discoidal particle in an extended helical conformation wherein the helical axes are oriented perpendicular to the plane of the phospholipid bilayer [26]. The rHDL/res particles resemble rHDL in geometry and size (discoidal, <20 nm diameter), with apoE3-NT likely oriented in a similar manner. Interestingly, both non-denaturing PAGE and TEM indicate that rHDL/res particles are more homogenous compared to rHDL. Based on the assumption that apoE3-NT adopts a fully extended α-helical structure, it is estimated to be ~30 nm long (191 residues x 0.15 nm rise per residue). In support of this estimation, a particle diameter of ~ 19 nm (with a perimeter of ~60 nm) would accommodate two apoE3-NT molecules in their fully extended forms, with a total of ~ 4 molecules present on each particle. Further, from the compositional analysis it appears that each particle can accommodate up to 4–6 resveratrol molecules.

The presence of resveratrol in rHDL did not alter the sLDLr binding ability of apoE3 as initially observed in co-IP assay. The sLDLr construct serves as a 'mini receptor' recapitulating the essential structural and functional features of the intact receptor [31]. The NT domain of apoE3 undergoes a dramatic conformational change upon lipid interaction, which presents apoE3 in a conformation that is competent to interact with the LDLr. The lipid-bound organization involves juxtaposition of two apoE3 molecules with neighboring receptor-binding epitopes, thereby creating a multivalent ligand [31]. Our studies indicate that the presence of resveratrol did not interact with the receptor-binding sites of apoE3 or affect the presentation of the multivalent sites. This was independently confirmed in glioblastoma cells, which over expresses LDLr with an estimated copy number of ~ 923,000 per cell [43, 44]. In this case, we examined the cellular uptake of rHDL/res at 37°C by following the uptake of the lipid components using DiI-labeled rHDL/res and of apoE3 by immunofluorescence. For the former, DiI-labeled commercial LDL and DiI/ rHDL were used as controls. In all cases, including those involving rHDL/res/DiI, DiI accumulation was observed around the nucleus as punctate endocytic vesicles. Similar observations were made when cellular uptake of rHDL/res was followed by immunofluorescence using apoE3 antibodies. Together, these studies show that the presence of resveratrol in rHDL did not affect the LDLr-mediated endocytosis process.

We confirmed that the internalization of rHDL/res is via the LDLr pathway using: (i) excess LDL as a competitor for LDLr binding and (ii) suramin, a polysulfonated naphthylamine derivative that is a known inhibitor of LDLr binding. Previous studies indicate that other receptors in the LDLr family such as the LDLr-related protein (LRP) [45] are abundant in cancer cell lines [46]. Whereas LDL is not a ligand for LRP, apoE is a well-established ligand, and plays an important role in binding and uptake of apoE containing lipoproteins[47, 48] especially in the central nervous system. The data suggest that LRP is likely not involved in rHDL/res uptake since no DiI fluorescence was noted in the presence of excess LDL.

Lastly, the presence of NBD on resveratrol did not affect its: (i) partitioning into rHDL, (ii) LDLr binding ability of rHDL/res/NBD, or, (iii) cellular LDLr-mediated uptake and internalization of rHDL/res/NBD. Interestingly, no significant intra-cellular fluorescence was noted when the cells were treated with res/NBD in DMSO in the absence of carrier vehicle at levels used in rHDL/res/NBD. Together, these studies indicate that resveratrol is internalized along with the hydrophobic lipid moiety in rHDL in a targeted manner.

**Fig 8:**
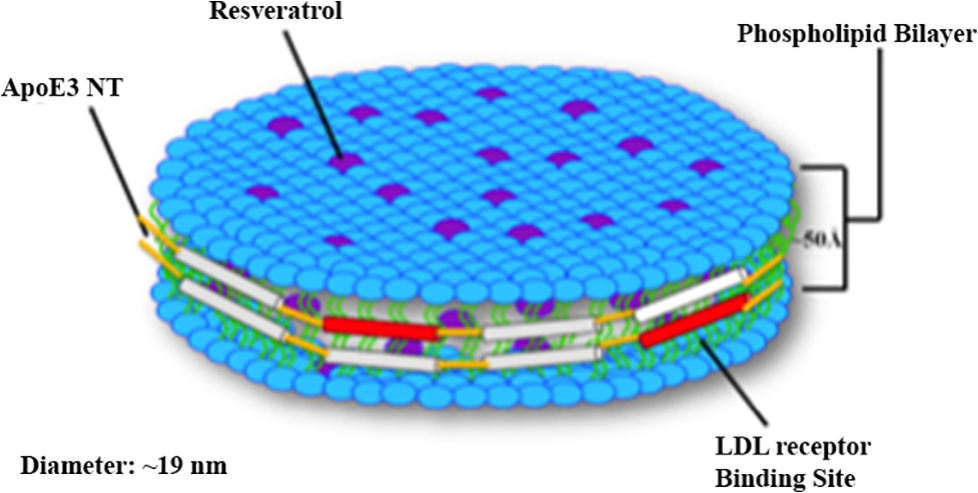
Model of apoE3 containing rHDL resveratrol embedded in the hydrophobic milieu. The phospholipid bilayer with embedded resveratrol is surrounded by the amphipathic α-helices of apoE3.

It is envisaged that resveratrol is embedded in the phospholipid bilayer circumscribed by apoE3 in an extended helical conformation, Fig 8. When consumed as a pure compound, the bioavailability of resveratrol is generally low because it is metabolized in the liver and gastrointestinal tract. However, rHDL offers a stable microenvironment for the embedded resveratrol, shielding it from degradation and therefore potentially leading to enhanced bioavailability. Other groups have shown that resveratrol afforded more protection against oxidative damage when incorporated into liposomes, compared to free resveratrol, with the liposomes likely preventing the cytotoxic effects of high resveratrol concentrations [49]. Whereas liposomes are eliminated relatively rapidly from the blood [50], nanoparticles appear to be more stable in blood and less biodegradable [51]. In addition, solid lipid nanoparticles (<180 nm) bearing resveratrol were shown to move rapidly across cell membrane in keratinocyte cell lines and distribute in the cytosol [52]. The smaller size of the rHDL/res compared to liposomes (25 nm—2.5 μm) and micelles (10–100 nm) appears to be an additional advantage as a drug delivery system, since large carriers can cause problems such as embolism formation and limited diffusion in tissue [51].

The targeted delivery of resveratrol or other antioxidant or anti-inflammatory agents to intracellular sites such as the endosomes and lysosomes is significant since endocytic trafficking is implicated in development of disorders such as Alzheimer disease (AD) or Huntington's disease. One of the characteristic features of AD is accumulation of amyloid β (Aβ) plaque in the brain [53], with endosomes serving as one of the main sites for generating Aβ. Several reports indicate that abnormal endocytic trafficking may play a role in the etiology of AD [54, 55]. Since resveratrol has anti-amyloid properties and appears to inhibit aggregation of Aβ that induce neuronal apoptosis [56, 57], the current studies are relevant in terms of our ability to deliver resveratrol to endosomes and lysosomes.

## Supporting Information

S1 File Fig A. Density gradient ultracentrifugation of rHDL and rHDL/res. rHDL (*left column*) or rHDL/res (*right column*) were separated from lipid-free protein and protein-free lipid by KBr density gradient ultracentrifugation. The presence of protein in each fraction was determined by BCA assay (*Panels A and B*) and by SDS-PAGE analysis (*Panels C and D*), and the presence of lipids using the Phospholipid C assay kit (*Panels E and F*). **Fig B. RP-HPLC profile of free resveratrol (A) and rHDL/res and rHDL (B). Fig C.** ^**1**^ **H NMR (300 MHz) of res/NBD in Acetone-D6**. **Fig D.** ^**13**^ **C NMR (75 MHz) of res/NBD in Acetone-D6. Fig E. Distortionless Enhancement by Polarization Transfer (DEPT)-135 NMR of res/NBD in Acetone-D6. Fig F. Mass spectrum of res/NBD. [M+H]** ^**+**^ **at 477.**
(PDF)
