# Supplementary material for: Targeted Intracellular Delivery of Resveratrol to Glioblastoma Cells Using Apolipoprotein E-Containing Reconstituted HDL as a Nanovehicle
Source: PLoS One. 2015 Aug 10;10(8):e0135130. doi: 10.1371/journal.pone.0135130 (PMC4530947; doi:10.1371/journal.pone.0135130)
Supplement: S1 File — rHDL (left column) or rHDL/res (right column) were separated from lipid-free protein and protein-free lipid by KBr density gradient ultracentrifugation. The presence of protein in each fraction was determined by BCA assay (Panels A and B) and by SDS-PAGE analysis (Panels C and D), and the presence of lipids using the Phospholipid C assay kit (Panels E and F). Fig B. RP-HPLC profile of free resveratrol (A) and rHDL/res and rHDL (B). Fig C. 1 H NMR (300 MHz) of res/NBD in Acetone-D6. Fig D. 13 C NMR (75 MHz) of res/NBD in Acetone-D6. Fig E. Distortionless Enhancement by Polarization Transfer (DEPT)-135 NMR of res/NBD in Acetone-D6. Fig F. Mass spectrum of res/NBD. [M+H] + at 477. (PDF) [file pone.0135130.s001.pdf]

Supporting Information

Targeted Intracellular Delivery of Resveratrol to Glioblastoma Cells using Apolipoprotein E-Containing Reconstituted HDL as a Nanovehicle

Separation of rHDL/res from lipid-free apoE3-NT

Resveratrol was incorporated into rHDL preparations during the reconstitution process. Reconstitution was performed with purified apoE3-NT and DMPC in the absence or presence of resveratrol, followed by density gradient ultracentrifugation to separate lipoprotein fractions from lipid-free protein and protein-free lipids. **S1 Figure A** shows the protein and lipid profile of the various fractions, #1 being the top fraction. The presence of apoE3-NT in each fraction was determined by protein assay (**Panels A and B**) and SDS-PAGE analysis (**Panels C and D**), the presence of DMPC was determined by the Phospholipid C assay kit (**Panels E and F**), both in the absence (**Panels A, C and E**) and presence (**Panels B, D and F**) of resveratrol. Fractions 2, 3, and 4 were enriched in apoE3-NT and phospholipids in the absence or presence of resveratrol, indicative of lipoprotein complex formation. These fractions were pooled and referred to as rHDL and rHDL/res, respectively.

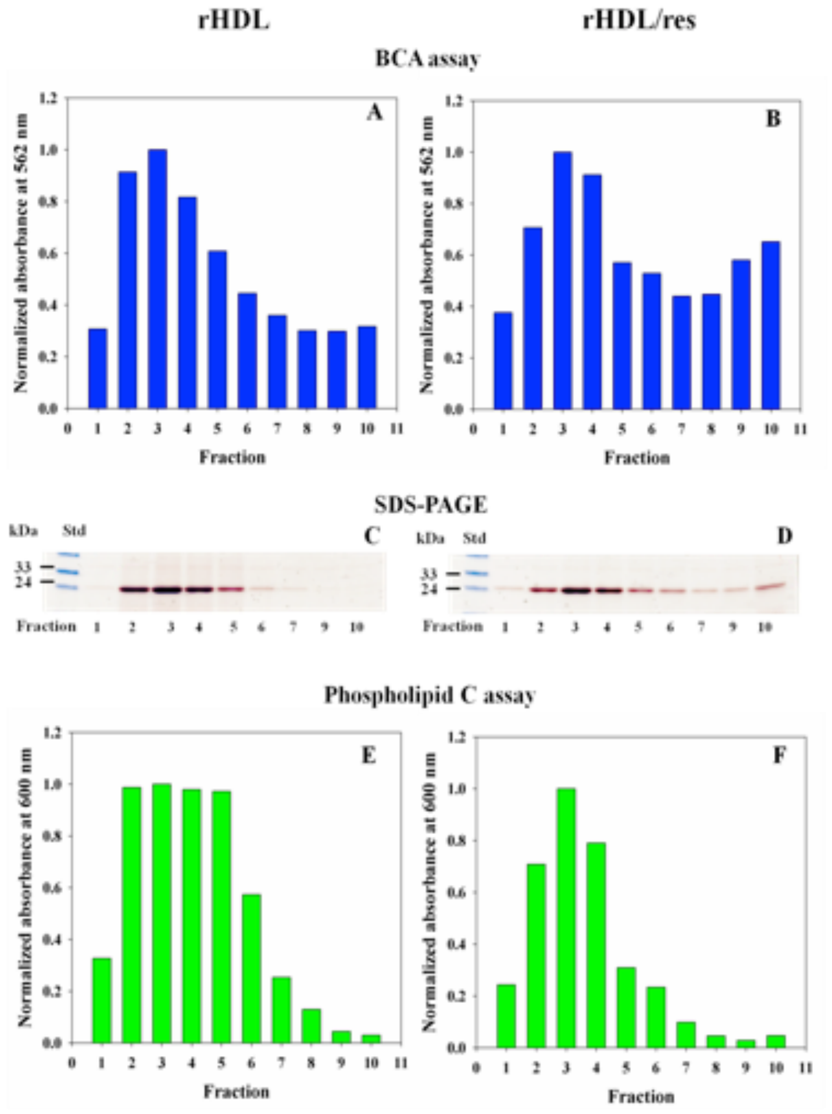

S1 Figure A. Density gradient ultracentrifugation of rHDL and rHDL/res. rHDL (*left column*) or rHDL/res (*right column*) were separated from lipid-free protein and protein-free lipid by KBr density gradient ultracentrifugation. The presence of protein in each fraction was determined by BCA assay (*Panels A and B*) and by SDS-PAGE analysis (*Panels C and D*), and the presence of lipids using the Phospholipid C assay kit (*Panels E and F*).

Reversed Phase High Performance Liquid Chromatography (RP-HPLC)

To determine the amount of resveratrol incorporated into rHDL, RP-HPLC (Beckman System Gold HPLC Model 166 UV/Vis detection) was performed. A stock solution of 0.03 mg/ml of resveratrol in sterile water was diluted to 0.005, 0.01, 0.015, 0.02 and 0.03 mg/ml and used as standards. The concentrations of rHDL and rHDL/res solutions were adjusted to 0.1mg/ml protein. The samples (50 µl,) were injected in duplicate into the HPLC column (Thermo Scientific Hypersil GOLD C18 250 x 4.6 mm ID 5 µm, Part #: C-25005-254630), and eluted with an acetonitrile gradient (A: water/acetonitrile/acetic acid mixture (75/24.9/0.1 (v/v); B: acetonitrile/acetic acid mixture (99.9/0.1 (v/v)). The flow rate was maintained at 1.0 ml/min for a run time of 50 min and the eluted samples monitored at 310 nm. Quantification was based on the peak area with results integrated and displayed by a 32-Karat Beckman Gold software (version 3.0).

Resveratrol elutes with a retention time of 20 min, with a minor peak at 23 min (**S1 Figure B**, Panel A). The HPLC profile of rHDL alone showed no major peak during the 50 min period (Figure *S1,* Panel B). On the other hand, rHDL/res showed a major peak with retention time of 17 min and a minor peak at 21 min (**S1 Figure B**, Panel B), qualitatively resembling the profile of resveratrol (**S1 Figure B**, *Panel A*). While the major peak represents *trans*-resveratrol, the minor likely represents *cis*-resveratrol. [1] The reason for the earlier elution time for resveratrol in rHDL/res is not known. Nevertheless, the amount of resveratrol in rHDL/res was determined to be 167 µM based on the standard curve obtained from a plot of resveratrol concentration versus area under the curve for the major peak.

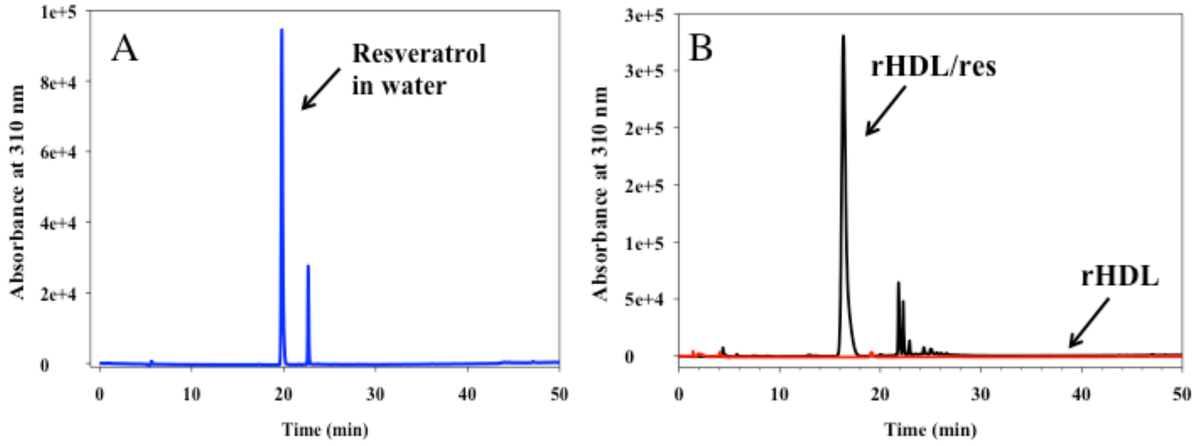

S1 Figure B. RP-HPLC profile of free resveratrol (Panel A) and rHDL/res and rHDL (Panel B).

Chemical synthesis of res/NBD

*Synthesis of **B***

Slurry of 0.72 g (3.15 mmoles) resveratrol, **A**, and 0.87 g (6.30 mmoles, 2 eq) K_2CO_3 in 20 mL acetone was stirred at room temperature for 15 min. Then, 0.39 mL (6.30 mmoles, 2 eq) methyl iodide was added and the mixture was stirred at 50 °C for 48 h. When cooled to room temperature, solvent was removed by rotary evaporation and the residue was partitioned between 1M HCl (20 mL) and EtOAc (20 mL). The aqueous fraction was further extracted with EtOAc (2x10 mL) and the combined EtOAc fraction was washed with water. Drying of organic phase over anhydrous $MgSO_4$ followed by solvent removal afforded a brown viscous liquid, which was subjected to flash chromatography (Hexane: EtOAc = 7:3) to get 0.27 g pure product as a white solid. Mixture of some unreacted resveratrol and monomethoxy product was also isolated from the column, which was further reacted following the similar procedure to get 0.13 g pure product. Total amount obtained = 0.4 g (50% yield). ^1H NMR (300 MHz, $CDCl_3$, 298 K) ∂ ppm 3.81 and 3.83 (2xs, 6H), 5.04 (s, 1H), 6.31 (t, J = 2.4 Hz, 1H), 6.58-6.65 (m, 2H), 6.81-7.04 (m, 4H), 7.38-7.45 (m, 2H).

*Synthesis of **C***

A mixture of 0.2 g (0.78 mmoles) **B**, 0.32 g (2.34 mmoles, 3 eq) K_2CO_3 and 0.17 g (1.17 mmoles, 1.5 eq) 2-chloro-N,N-dimethylethyleneamine hydrochloride in 10 mL dry acetone under nitrogen atmosphere was stirred overnight at refluxing condition. When TLC indicated the complete consumption of **B**, the mixture was allowed to cool to room temperature, solvent was removed by rotary evaporation and the solid was partitioned between 20 mL $CHCl_3$ and 20 mL 0.1 M HCl. The aqueous fraction was further extracted with $CHCl_3$ (2x10 mL) and the combined $CHCl_3$

fraction was washed with water (2x20 mL). Drying of organic fraction over anhydrous $MgSO_4$ followed by rotary evaporation of the solvent yielded 0.23 g off-white solid as a pure product (92% yield). ^{1}H NMR (300 MHz, MeOH-d4, 298 K) ∂ ppm 3.00 (s, 6H), 3.61 (t, J = 5.1 Hz, 2H), 3.81 and 3.82 (2xs, 6H), 4.38 (t, J = 5.1 Hz, 2H), 6.48 (t, J = 2.1 Hz, 1H), 6.77-6.81 (m, 2H), 6.90-6.99 (m, 3H), 7.11 and 7.17 (2xs, 1H) 7.47-7.50 (m, 2H).

*Synthesis of **D***

Under nitrogen atmosphere, a mixture of 0.15 g (0.45 mmoles) **C** and 0.058 mL (0.75 mmoles, 1.65 eq) methyl chloroformate in 5 mL 1,2-dichloroethane was stirred at refluxing condition for 5 h. Then, additional 0.058 mL methyl chloroformate was added and the mixture was stirred at refluxing condition overnight. When cooled to room temperature, the reaction mixture was diluted by 20 mL $CHCl_3$ and washed with 0.1 M NaOH. The aqueous fraction was back extracted with $CHCl_3$ (2x20 mL). The combined $CHCl_3$ fraction was washed with water and dried over anhydrous $MgSO_4$. Removal of the solvent using rotary evaporation afforded the desired product as a colorless viscous liquid (0.12 g, 82% yield). ^{1}H NMR (300 MHz, $CDCl_3$, 298 K) ∂ ppm 2.37 (s, 4H), 2.77 (t, J = 5.7 Hz, 2H), 3.82 and 3.83 (2xs, 6H), 4.11 (t, J = 6.0 Hz, 2H), 6.40 (t, J = 2.4 Hz, 1H), 6.64-6.68 (m, 2H), 6.86-6.92 (m, 3H), 7.01 and 7.06 (2xs, 1H), 7.43-7.46 (m, 2H).

*Synthesis of **E (res/NBD)***

To a mixture of 0.1 g (0.32 mmoles) **D** and 0.07 g (0.35 mmoles, 1.1 eq) NBD-chloride in 6 mL acetonitrile under nitrogen atmosphere was added 0.067 mL (0.48 mmoles, 1.5 eq) triethyl amine, and the mixture was stirred at 60 °C overnight. When TLC indicated the complete consumption

of the starting material, the reaction mixture was cooled to room temperature and the solvent was removed by rotary evaporation. The residue was taken up in 15 mL EtOAc, washed with saturated $NaHCO_3$ solution (2x 10 mL) followed by water (10 mL), and dried over anhydrous $MgSO_4$. Removal of the solvent afforded dark red oily residue, which was subjected to flash chromatography (Hexane: EtOAc = 2:1) to get the pure product **E** (0.047 g, 31% yield). ^{1}H NMR (300 MHz, Acetone-d6, 298 K) ∂ ppm 3.63 (bs, 3H), 3.76 and 3.80 (2xs, 6H), 4.47 (t, J = 5.4 Hz, 2H), 4.65 (t, J = 4.2 Hz, 2H), 6.36 (t, J = 2.1 Hz, 1H), 6.49 (d, J = 9.0 Hz, 1H), 6.71 (s, 2 H), 6.90-6.99 (m, 3H), 7.11 and 7.16 (2xs, 1 H), 7.47-7.50 (m, 2H), 8.48 (d, J = 9.3 Hz, 1H). ^{13}C NMR (75 MHz, Acetone-d6, 298 K) ∂ ppm 42.97, 55.49, 55.57, 55.61, 66.86, 100.81, 103.10, 105.45, 105.59, 114.93, 122.91, 126.98, 128.64, 129.59, 130.00, 130.73, 136.40, 140.81, 145.86, 146.93, 160.49, 160.69, 162.01. The calculated mass for $C_{25}H_{24}N_4O_6$ was 476.17, and the observed mass was $[M+H]^+$ 477.

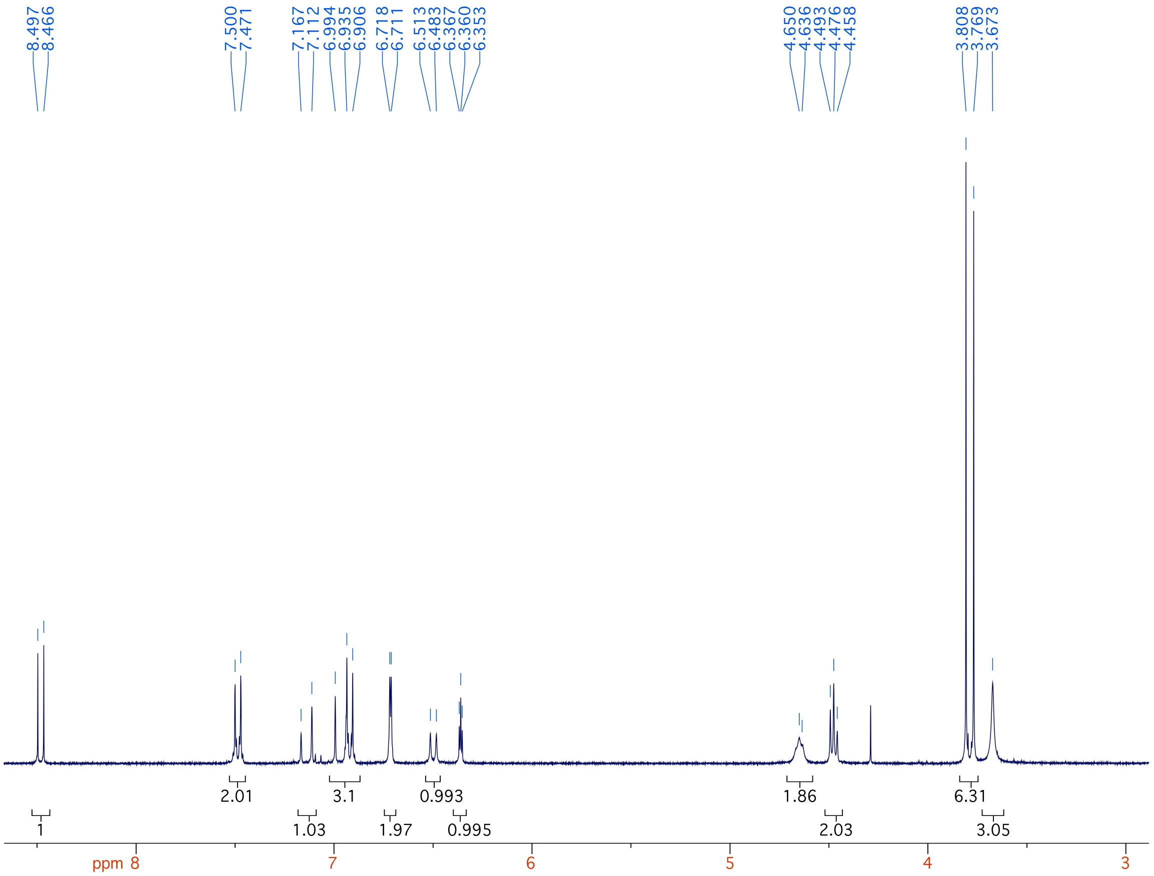

S1 Figure C. ^{1}H NMR (300 MHz) of res/NBD in Acetone-D6.

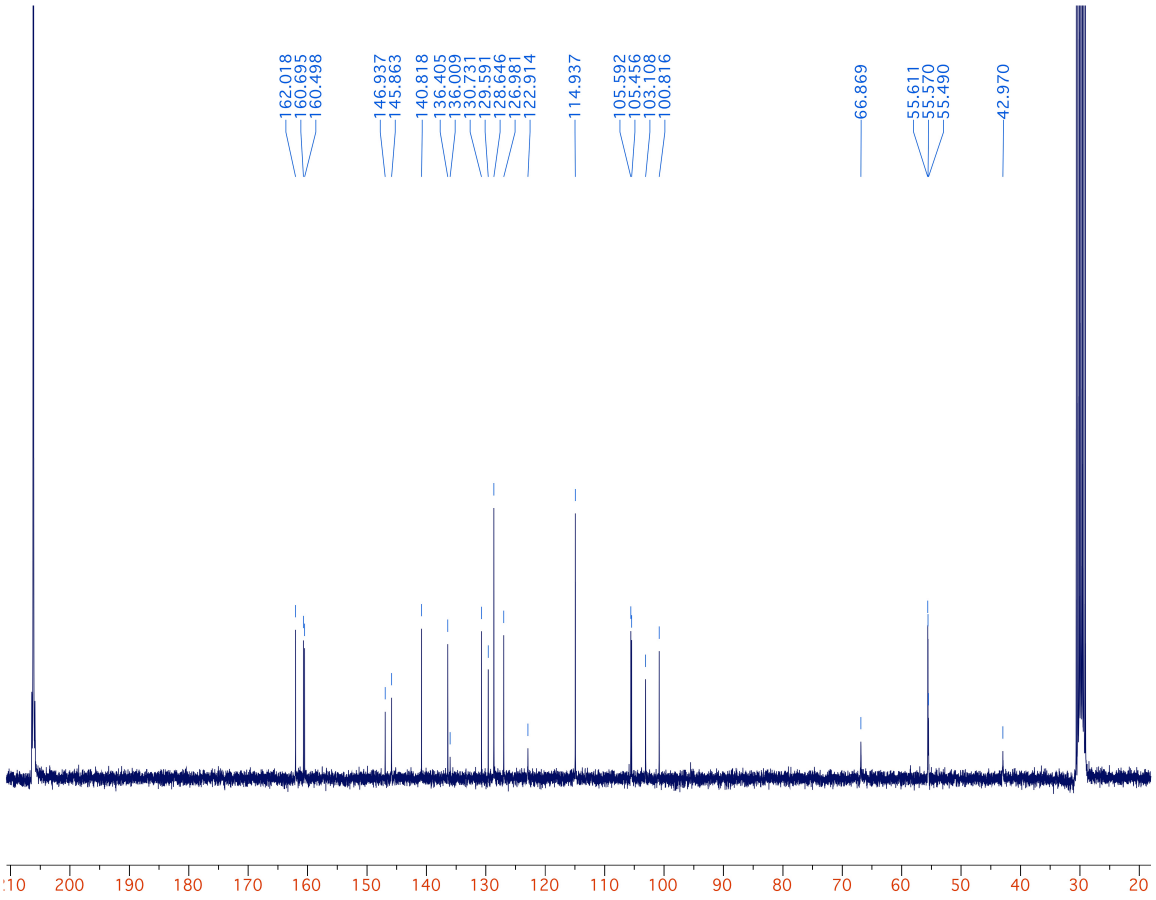

S1 Figure D. ^{13}C NMR (75 MHz) of res/NBD in Acetone-D6.

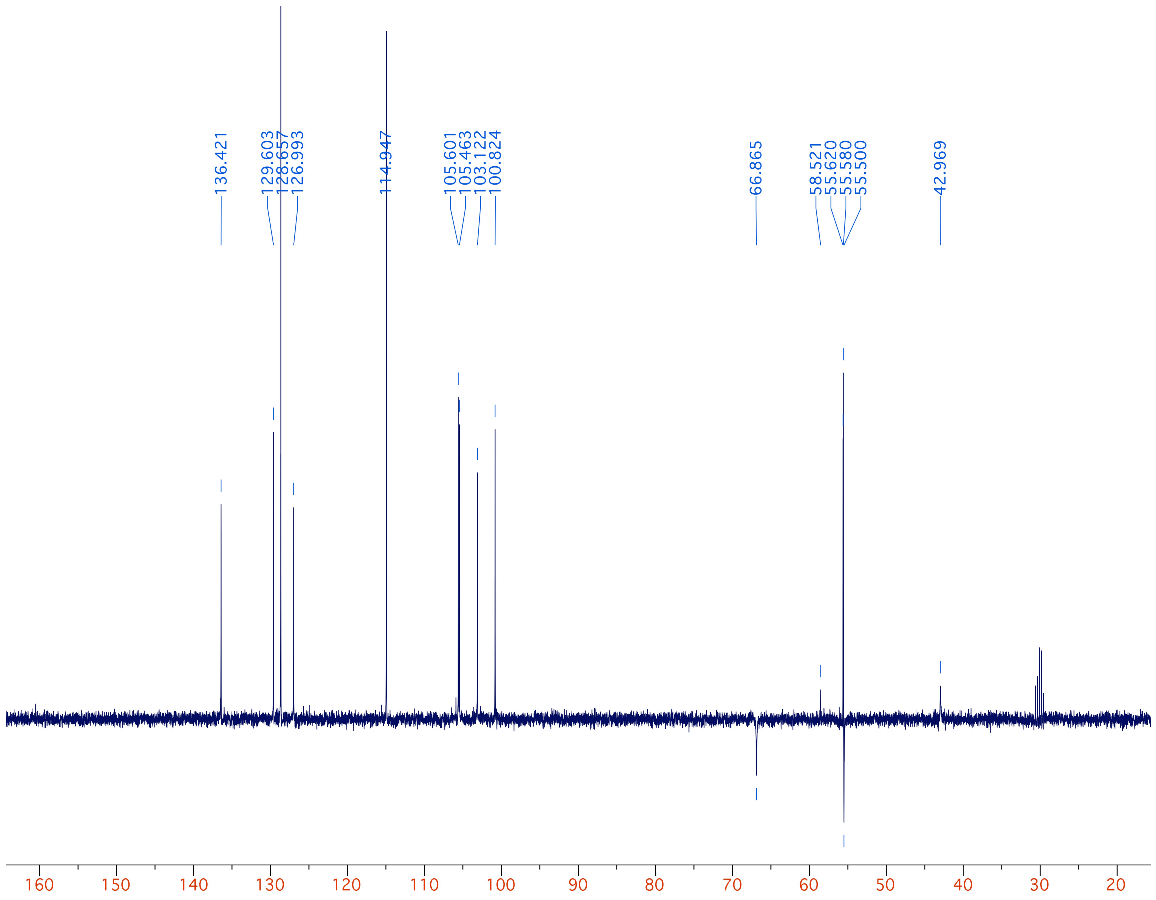

S1 Figure E. Distortionless Enhancement by Polarization Transfer (DEPT)-135 NMR of res/NBD in Acetone-D6.

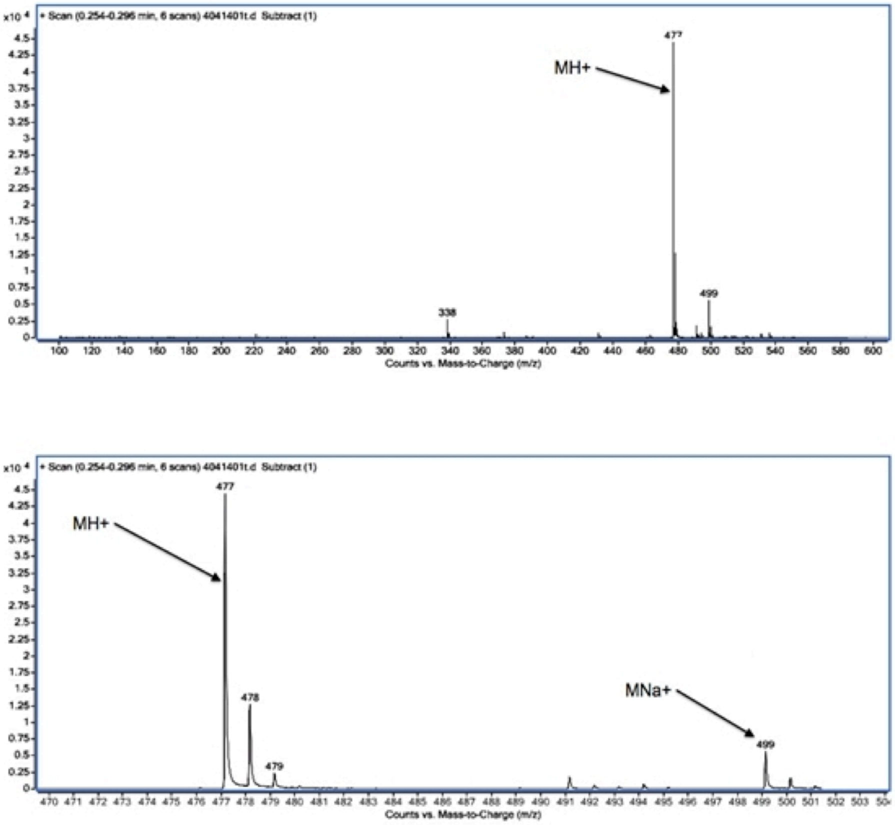

S1 Figure F. Mass spectrum of res/NBD. $[M+H]^+$ at 477.

References

1. Figueiras, T. S.; Neves-Petersen, M. T.; Petersen, S. B. Activation energy of light induced isomerization of resveratrol. *J Fluoresc* 2011;**21**:1897-906
